# Comparison of effect between nasobiliary drainage and biliary stenting in malignant biliary obstruction: a systematic review and updated meta-analysis

**DOI:** 10.1186/s12957-020-01848-1

**Published:** 2020-04-10

**Authors:** Wei Zhang, Xu Che

**Affiliations:** 1 Department of Pancreatic and Gastric Surgery, National Cancer Center/National Clinical Research Center for Cancer/Cancer Hospital, Chinese Academy of Medical Sciences and Peking Union Medical College, 17 Panjiayuan Nanli, Chaoyang District, Beijing, 100021 China; 2 Department of Hepatobiliary and Pancreatic Surgery, National Cancer Center/National Clinical Research Center for Cancer/Cancer Hospital & Shenzhen Hospital, Chinese Academy of Medical Sciences and Peking Union Medical College, Shenzhen, 518116 China

**Keywords:** Endoscopic biliary stenting, Endoscopic nasobiliary drainage, Malignant biliary obstruction, Preoperative biliary drainage

## Abstract

**Background:**

To compare the efficacy of endoscopic nasobiliary drainage (ENBD) and endoscopic biliary stenting (EBS) in preoperative biliary drainage (PBD).

**Methods:**

ENBD and EBS related literature of patients with malignant biliary obstruction published before September 2019 were collected from PubMed, EMBASE, and Cochrane Library for comparison analysis. Revman 5.3 statistical software was used for analysis.

**Results:**

Nine studies were used for our comparative study. A total of 1435 patients were included, which consisted of 813 in the ENBD group and 622 in the EBS group. Meta-analysis showed that patients with malignant biliary obstruction who received ENBD had reductions in the rates of preoperative cholangitis (RR  =  0.46, 95% CI  =  0.34–0.62, *P*  <  0.00001), preoperative pancreatitis (RR  =  0.69, 95% CI  =  0.50–0.95, *P*  = 0.02), stent dysfunction (RR  =  0.58, 95% CI  =  0.43–0.80, *P*  = 0.0008), morbidity (RR  =  0.77, 95% CI  =  0.64–0.93, *P*  =  0.007), and postoperative pancreatic fistula (RR  =  0.65, 95% CI  =  0.45–0.92, *P*  =  0.02) compared with patients who received EBS.

**Conclusions:**

The rates of preoperative cholangitis, preoperative pancreatitis, post-operative pancreatic fistula, stent dysfunction, and morbidity of ENBD patients were lower than those of EBS patients. In clinical practice, the physical condition of each patient and their tolerance should be fully considered. ENBD should be given priority. EBS should be replaced if stent dysfunction or intolerance occurs.

## Introduction

Malignant biliary obstruction (MBO) is a large group of malignant tumors that cause biliary obstruction, including hilar cholangiocarcinoma, pancreatic head cancer, and cholangiocarcinoma. Surgical radical resection is the only treatment that could cure and obtain a long-term survival rate, and the majority of patients need combined hepatectomy to achieve a radical cure [1]. Most patients with MBO diseases have obstructive jaundice of varying degrees. Preoperative biliary drainage (PBD) is needed to improve liver function, coagulation function, nutritional status, and immune function in order to avoid acute cholangitis and promote liver regeneration, as well as reducing the risk of operative and postoperative complications [2–4]. Common PBD methods include endoscopic biliary stent (EBS), endoscopic nasobiliary drainage (ENBD), and percutaneous transhepatic biliary drainage (PTBD). Each PBD has a certain risk of complications which can endanger life and result in a lost opportunity for an operation. It has been reported that PBD can cause complications such as biliary hemorrhage, acute cholangitis, and acute pancreatitis, with an incidence of 8%. In addition, PBD can also cause bacterial contamination of bile, and therefore increases the probability of infection after an operation, and even metastasis of tumors [5–7]. PTBD belongs to invasive drainage, which can be placed with multiple drainage tubes to relieve jaundice. However, PTBD is not considered as the first choice due to its increased probability of tumor metastasis by the invasive procedure. For an easy-to-operate drainage EBS and ENBD, it is still not clear which one has the best preoperative effect to reduce jaundice. Multicenter large sample randomized controlled clinical trials are still needed. Therefore, we systematically reviewed relevant studies since the emergence of EBS and ENBD, and made a quantitative analysis to explore their implementation effect, application value, and mode selection in order to provide an evidence-based reference for clinical practice.

## Methods

### Retrieval strategy

**Fig. 1:**
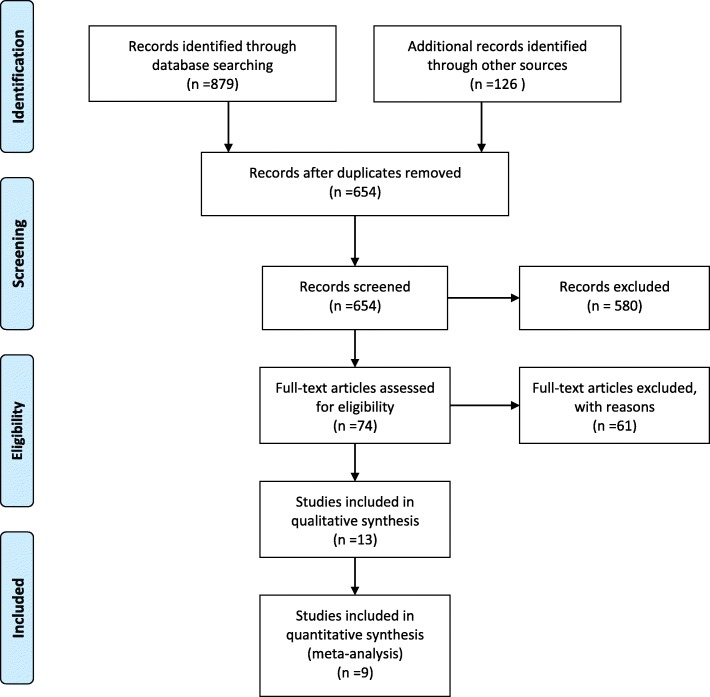
PRISMA 2009 flow diagram of literature screening

The literature was retrieved from PubMed, EMBASE, and Cochrane Library databases using the keywords “nasobiliary drainage,” “nasobiliary catheter,” “nasobiliary drain,” and “ENBD,” and the combination of “internal endoscopic biliary drainage,” “internal EBD,” “endoscopic biliary stenting,” “EBS,” “endoscopic retrograde biliary drainage,” “ERBD,” “stent,” and “stenting” in order to identify relevant studies published before September 2019. Figure 1 summarizes the process for retrieving relevant literature, and Table 1 summarizes the patient characteristics and surgical results included in the study.

**Table 1:** Basic characteristics and quality assessment of enrolled documents

	Country	Study design	Period of study	Type of PBD	Case	Sex (M/F)	Age	Quality (NOS)
Fujii T [8]	Japan	PC	2008-2014	ENBD	50	30/20	66.5 (39-83)	7
				EBS	72	46/26	67 (38-84)	
Huang X [9]	China	RC	2005-2014	ENBD	18	5/13	60.6 ± 8.4	7
				EBS	37	10/27	58.1 ± 8.3	
Jo JH [10]	Korea	RC	2005-2012	ENBD	13	8/13	58.9 (42-77)	5
				EBS	42	23/19	61.1 (29-80)	
Kawakami [11]	Japan	RC	1999-2009	ENBD	60	44/16	71 (45–81)	7
				EBS	20	4/16	70 (59–77)	
Kawakubo [12]	Japan	RC	2009-2014	ENBD	85	74/44	69 ± 9	6
				EBS	33			
Nakai Y [13]	Japan	RC	2010-2014	ENBD	281	189/92	71 (64-76)	7
				EBS	76	47/29	70 (65-74)	
Sasahira [14]	Japan	RC	2010-2012	ENBD	166	64/102	70 (63-76)	7
				EBS	253	64/189	69 (62-75)	
Sugiyama [15]	Japan	RC	2008-2012	ENBD	38			6
				EBS	38			
Zhang G [16]	China	RC	2009-2016	ENBD	102	58/44	55.26 ± 9.07	7
				EBS	51	29/22	56.24 ± 9.65	

*EBS* endoscopic biliary stenting, *ENBD* endoscopic nasobililary drainage, *NA* not available, *NOS* Newcastle-Ottawa Score, *RC* retrospective clinical study, *PC* prospective clinical study

### Inclusion criteria

Inclusion criteria were as follows: (1) study subjects: the cases with confirmed MBO, feasible limited operation; (2) original published literature which contained comparison research on the efficacy of ENBD and EBS, including randomized controlled studies, prospective observational studies, or retrospective observational studies; (3) the sample size of the study: unlimited; (4) follow-up time: more than 3 months; (5) the language of published literature: English; (6) research type: human studies; (7) research indicators: incidence rates of preoperative cholangitis, preoperative pancreatitis, stent dysfunction, morbidity, and postoperative pancreatic fistula.

### Data exclusion and quality assessment

Articles that conformed to the following rules were removed: (1) studies with incomplete information, inability to extract valid data, unresponsive contact with authors, repeated publication, unpublished follow-up, and unknown follow-up time; (2) one-arm study of ENBD or EBS, studies reporting outcomes about ENBD or EBS alone without comparison; (3) MBO cases that lost the opportunity of surgical treatment; (4) literature written in languages other than English; (5) robotic research, reviews, case reports, and animal experiments.

This meta-analysis only enrolled 9 cohort studies (CSs). CSs were graded according to the Newcastle-Ottawa Scale (NOS), including selection, comparability, exposure evaluation, or outcome evaluation. NOS adopted the semi-quantitative principle of star system to evaluate the quality of literature, which is divided into 9 stars (Additional file 1 Appendix file 2).

### Statistical analysis

Meta-analysis was performed by Review Manager 5.3 software. Risk ratio (RR) was selected for dichotomous data. Weighted mean difference (WMD) and its 95% confidence interval (CI) were selected for continuous data. Chi-square test was used for the homogeneity test of each study. The fixed effect model was used only if homogeneity was accepted (*I*^*2*^ < 35%, *P* < 0.05). If there was significant clinical heterogeneity among the studies, the random effect model was used. *P* values < 0.05 were considered as statistically significant. Sensitivity analysis was performed by removing 1 study at a time to assess whether the results could have been markedly affected by a single study. Subgroup analysis was performed according to the factors affecting the outcome. The funnel plots were qualitatively used to judge whether there was publication bias in these studies. Begg’s test and Egger’s test were quantitatively used to evaluate the publication bias of the included study, as shown in Table 2. The significance level was limited to 0.05.

**Table 2:** Meta-analysis results of all available studies in measured outcomes

Measured outcomes	Subgroup	No. studies	No. patients	Heterogeneity test		Model	RR/WMD	95% CI	*P*	Begg’s test		Egger’s test
				*I*^*2*^(%)	*P*					Pr > |z|*	Pr > |z|**	P > |t|*
Preoperative cholangitis	Total	9	813 vs. 622	19.1	0.273	Random	0.46	0.34, 0.62	< 0.001	0.404	0.466	0.73
	HCC	2	145 vs. 53	81.5	0.02	Random	0.5	0.19, 1.3	0.156	--	--	--
	DBO	4	535 vs. 439	17.4	0.259	Random	0.38	0.26, 0.57	< 0.001	0.174	0.308	0.282
Preoperative pancreatitis	Total	7	750 vs. 508	0	0.929	Fixed	0.69	0.50, 0.95	0.023	0.881	1	0.551
	HCC	2	145 vs. 53	0	0.999	Fixed	0.67	0.30, 1.47	0.314	--	--	--
	DBO	3	485 vs. 367	0	0.825	Fixed	0.74	0.50, 1.12	0.152	0.602	1	0.603
Stent dysfunction rate	Total	6	464 vs. 437	38.6	0.149	Random	0.58	0.43, 0.8	0.001	0.188	0.26	0.311
	HCC	2	145 vs. 53	0	0.319	Random	0.48	0.35, 0.67	< 0.001	--	--	--
	DBO	2	204 vs. 291	26.3	0.244	Random	0.59	0.39, 0.90	0.015	--	--	--
Morbidity	Total	5	231 vs. 188	0	0.418	Fixed	0.77	0.64, 0.93	0.007	0.142	0.221	0.163
POPF	Total	3	170 vs. 160	7.7	0.338	Fixed	0.65	0.45, 0.92	0.016	0.602	1	0.536

*HCC* hilar cholangiocarcinoma; *DBO* distant biliary obstruction; *POPF* postoperative pancreatic fistula; *No.*number of; *RR* risk ratio; *WMD* weighted mean difference; *CI* confidence interval

*P value

**P value (continuity corrected)

--Not applicable

## Results

### Search results and study characteristics

Nine relevant publications were used in this study, 8 RCSs and 1 prospective cohort study. The cumulative sample size in these studies was 1435 patients, including 813 samples in the ENBD group and 622 samples in the EBS group. Basic characteristics and quality assessment of the enrolled documents are shown in Table 1. Meta-analysis results of endpoints from all available studies are shown in Table 2 at end of article.

### Incidence of preoperative cholangitis

**Fig. 2:**
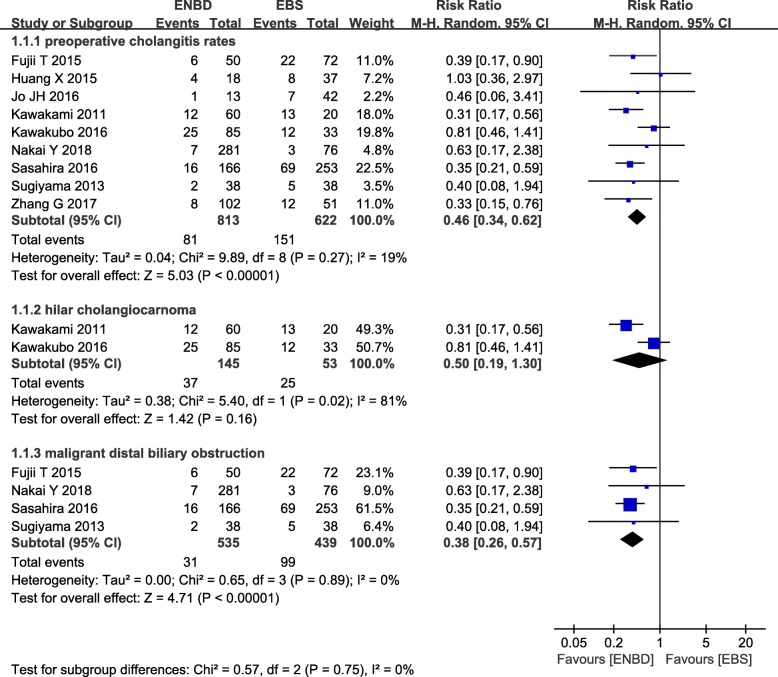
Forest plots of preoperative cholangitis rates

From the 9 studies, 813 cases in the ENBD group and 622 cases in the EBS group were used in this meta-analysis [8–16]. Low heterogeneity (*I*^2^  = 19%, *P*  = 0.27) was found, so we chose a random-effect model to pool the RR. Overall, the pooled data demonstrated that ENBD was associated with a low incidence of preoperative cholangitis (RR  =  0.46, 95% CI  =  0.34–0.62, *P*   <  0.00001) in the MBO patients. Subgroup analysis showed a higher incidence of preoperative cholangitis in the EBS group than in the ENBD group among hilar cholangiocarcinoma (HCC) patients (RR   =  0.50, 95% CI  =  0.19–1.30, *P* =  0.16) and malignant distal biliary obstruction patients (RR =  0.38, 95% CI  =  0.26–0.57, *P* <  0.00001) (Fig. 2 Additional file 1 Appendix file 4).

### Incidence of preoperative pancreatitis

**Fig. 3:**
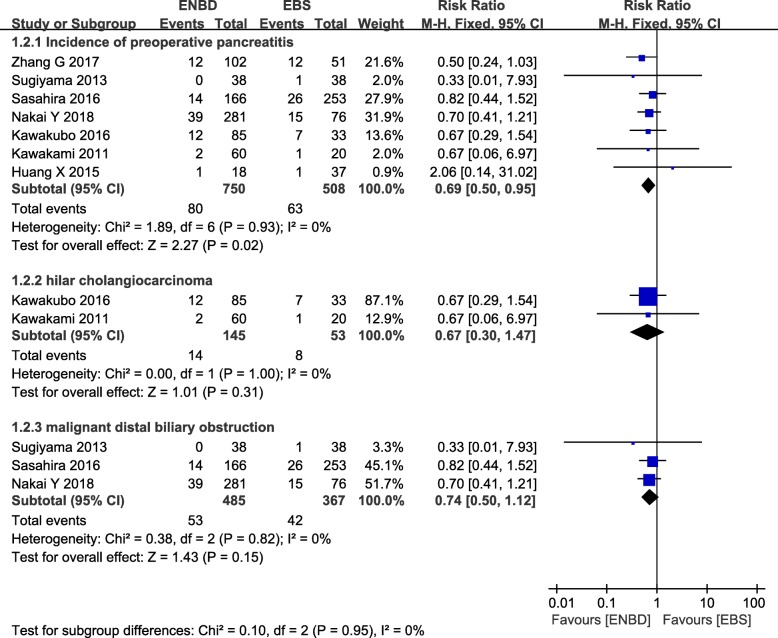
Forest plots of preoperative pancreatitis rates

From 7 studies, 750 cases in the ENBD group and 508 cases in the EBS group were used in this meta-analysis [8, 9, 11–16]. No heterogeneity (*I*^*2*^  =  0%, *P*  =  *0.93*) was found, so we chose a fixed-effect model to pool the RR. Overall, the pooled data demonstrated that ENBD was associated with a low incidence of preoperative pancreatitis (RR  =  0.69, 95% CI  =  0.50–0.95, *P*  = 0.02) in the MBO patients. Subgroup analysis showed there was no significant difference in the preoperative pancreatitis rate between ENBD and EBS in HCC patients (RR  =  0.67, 95% CI  =  0.30–1.47, *P*  =  0.31) or malignant distal biliary obstruction patients (RR  =  0.74, 95% CI  =  0.50–1.12, *P*  =  0.15) (Fig. 3 Additional file 1 Appendix file 5).

### Stent dysfunction rate

**Fig. 4:**
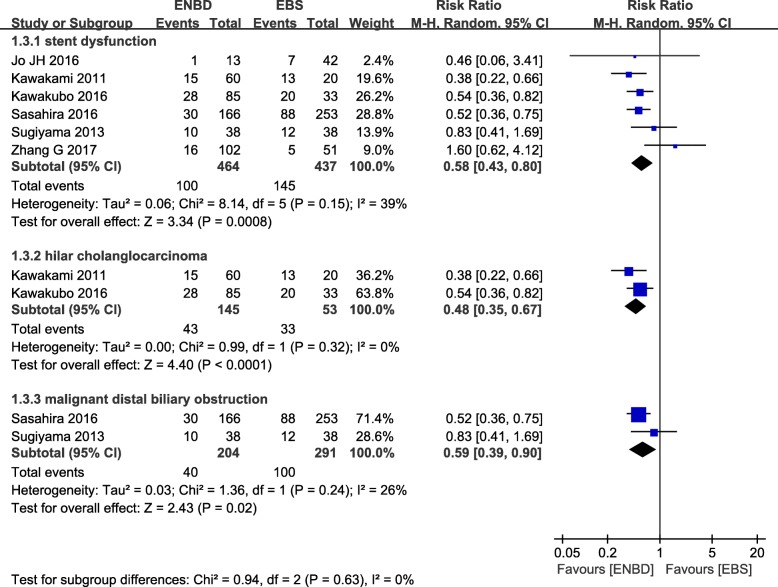
Forest plots of stent dysfunction rates

From 6 studies, 464 cases in the ENBD group and 437 cases in the EBS group reported a stent dysfunction rate [10–16]. Moderate heterogeneity (*I*^*2*^  =  39%*, P  =*  0.15) was found, so we chose a random-effect model to pool the RR. Overall, the pooled data demonstrated that ENBD was associated with a low incidence of stent dysfunction (RR  =  0.58, 95% CI  =  0.43–0.80, *P*  =  0.0008) in MBO patients. Subgroup analysis showed that the stent dysfunction rate was also higher in the EBS group than in the ENBD group among HCC patients (RR  =  0.0.48, 95% CI  =  0.35–0.67, *P*  <  0.0001) and malignant distal biliary obstruction patients (RR  =  0.59, 95% CI  =  0.39–0.90, *P*  =  0.02) (Fig. 4 Additional file 1 Appendix file 6).

### Morbidity

**Fig. 5:**
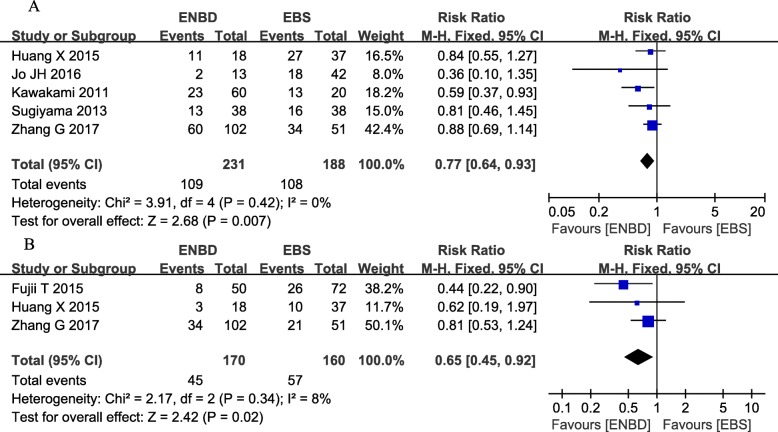
**a** Forest plots of morbidity rates. **b** Forest plots of pancreatic fistula rates

Five studies were used to assess morbidity, which was defined as the incidence of all pre- and postoperative complications [9–11, 14–16]. Although only 1 study showed that ENBD had a significant advantage in terms of morbidity compared with EBS, the pooled results had no heterogeneity (*I*^*2*^*=*  0%, *P  =*  0.42) and showed that ENBD had a significantly lower incidence of morbidity than EBS (RR  =  0.77, 95% CI  =  0.64–0.93, *P*  =  0.007) (Fig. 5a Additional file 1 Appendix file 7).

### Postoperative pancreatic fistula

Three studies were used to assess the rate of postoperative pancreatic fistula (POPF) [8, 9, 16]. The pancreatic fistula rate was significantly lower in the ENBD group than in the EBS group (RR  =  0.65, 95% CI  =  0.45–0.92, *P*  =  0.02) based on the pooled data, which showed low heterogeneity (*I*^*2*^*=*  8%, *P  =  0.34*) (Fig. 5b Additional file 1 Appendix file 8).

### Sensitivity analysis and assessment of risk of bias

**Fig. 6:**
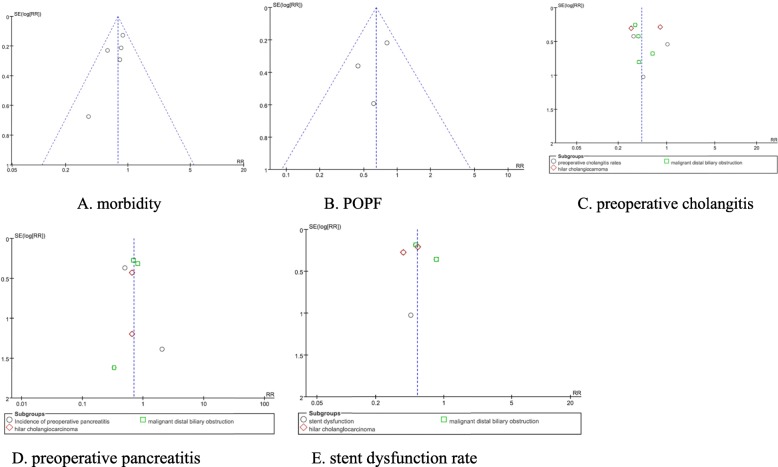
The funnel plots of meta-analysis

Sensitivity analysis suggested that the majority data in this meta-analysis were relatively stable. The funnel plots were used to judge whether there was publication bias in these studies. As shown in the funnel plots, the studies are basically symmetrical and the possibility of publication bias is low. No publication bias was detected by Begg’s test and Egger’s test. (Fig. 6 Additional file 1 Appendix file 9)

## Discussion

Malignant obstruction of the hepatobiliary system is caused by obstruction or compression of bile duct epithelial cancer, gallbladder cancer, pancreatic cancer, or metastatic cancer. The main clinical manifestations are jaundice, abdominal pain, and fever. A large amount of bile retention may cause liver dysfunction, and cause pancreatitis and cholangitis. Severe cases may also lead to complications such as sepsis and disseminated or diffuse intravascular coagulation (DIC), all of which may result in the loss of surgical intervention. Surgical resection is the only way to cure MBO. Surgery depends not only on the tumor itself but also on jaundice, liver function, the physical condition of the patient, and other complications [12]. Therefore, some patients must be treated with PBD to reduce jaundice in order to make surgery tolerable. Previous treatment for obstructive jaundice caused by MBO often used PTBD for biliary drainage [17, 18]. In recent studies, surgeons have preferred endoscopic biliary drainage to PTBD, taking into consideration the quality of life for patients and avoiding the spread of tumors and serious complications [19–21]. The operation of ENBD and EBS is relatively simple. At present, there is no randomized controlled clinical study comparing the clinical efficacy of these 2 PBD methods before radical resection of MBO cancer. Long patency drainage methods are often desired in patients who could not undergo surgery. For the temporary PBD before resecting MBO, the effect of drainage and the effect of PBD on the operation should be considered. In this study, the advantages and disadvantages of 2 kinds of drainage effects and complications were systematically discussed.

Inflammatory reactions including cholangitis and pancreatitis after post-ERCP are unavoidable in endoscopic biliary drainage [22, 23]. ENBD is an exogenous operation to drain bile and relieve biliary obstruction. It is convenient for biliary cytology and cholangiography [24]. However, long-term indwelling of a nasobiliary duct after ENBD may cause discomfort to laryngeal stimulation and disturbance of water and electrolyte, and there is a risk of the nasobiliary duct breaking or falling off due to the influence of hepatointestinal circulation. EBS, as an internal biliary drainage method, connects the biliary tract and duodenum with a stent without the abovementioned adverse effects. When EBS is used for distal malignant obstruction, the stent will obstruct due to the role of intestinal microorganisms, leading to food reflux. This is one of the causes of biliary tract infection and preoperative cholangitis, as well as one of the potential risks of complications of postoperative infection [8, 25].

Four studies included in this study had reported that the incidence of preoperative cholangitis in the ENBD group was significantly lower than that in EBS group. After combining the results, both total and hilar cholangiocarnoma subgroup analysis showed a lower preoperative incidence of cholangitis in ENBD, which was consistent with other studies [26, 27].

In addition, meta-analysis showed that the incidence of preoperative pancreatitis and stent dysfunction in the EBS group was higher than that in ENBD group, and the causes of the dysfunction were EBS stent occlusion and ENBD stent dislocation. Whether MBO biliary drainage is adequate or not is directly related to the degree of organ damage. Biliary obstruction and cholangitis due to poor biliary drainage will also have a great impact on the survival period and the quality of life of patients. Due to the advantages of rinsing in vitro, ENBD can effectively guarantee the smooth degree of drainage, prolong the survival time, and improve the quality of life for patients. Meta-analysis results provide evidence for this.

Pancreatic fistula, delayed gastric emptying, biliary fistula, and deep abdominal infection are the most common morbidities after pancreaticoduodenectomy (PD). Studies have shown that PBD causes bacterial translocation in the biliary tract, leading to cholangitis associated with this process, which makes the incidence of wound infection significantly higher than that of patients who did not receive PBD treatment before a pancreaticoduodenectomy (PD) [19, 20]. Fujii et al. also reported that the positive rate of bile or drainage fluid culture in the ERBD group was significantly higher than that in the ENBD group, and that the incidence of an abdominal abscess was significantly higher [8]. Zhang et al. did not find the difference of the overall complications of PD between the ENBD group and the EBS group; there was a significant difference in the incidence of deep abdominal infections but not in wound infections or pulmonary infections [16]. The evidence shows that the infection complications of PD are important factors affecting the treatment of EBD. Therefore, the reduction of morbidity of PD after an operation is an index to evaluate the efficacy of ENBD and EBS. In the enrolled literature, only Kawakami et al. suggested that the morbidity of ENBD was significantly lower than that of EBS [11], while other studies reported that there was no significant difference between the 2 groups. However, the pooled results showed that the incidence of ENBD was significantly lower than that of EBS.

Therefore, preoperative biliary drainage for MBO patients can give priority to ENBD. When stent dysfunction or intolerance occurs, the nasobiliary duct is replaced by a biliary stent, a so-called bridge PBD. Studies have shown that bridge PBD can shorten the length of the preoperative hospital stay and enables PBD to be carried out for a long time without aggravating the prognosis after PD [28].

## Conclusion

Regardless of the type of MBO, the rates of preoperative cholangitis, preoperative pancreatitis, post-operative pancreatic fistula, stent dysfunction, and morbidity of ENBD patients were lower than those of EBS patients. In clinical practice, the physical condition of each patient and their tolerance should be fully considered. ENBD should be given priority. EBS should be replaced if stent dysfunction or intolerance occurs.

## Limitation

There are several limitations in this article. First, most of the literature is a retrospective study. Although retrospective studies can reflect the application value of the real world, there may be selection bias in these non-randomized controlled studies. Second, since malignant biliary obstruction includes a variety of malignant tumors, although subgroup analysis is performed, its high level of mixed pathology may affect the applicability of this review.

## Supplementary information

**Additional file 1: Appendix file 1.** the Preferred Reporting Items for Systematic Reviews and Meta-Analyses (PRISMA) 2009 Checklist. **Appendix file 2.** the Risk of bias in the included retrospective cohort studies (by the Newcastle–Ottawa quality assessment tool). **Appendix file 3.** Meta-Analysis Results of All Available Studies in Measured Outcomes. **Appendix file 4.** the forest map of the incidence of incidence of preoperative cholangitis. **Appendix file 5.** the forest map of the incidence of incidence of preoperative pancreatitis. **Appendix file 6.** the forest map of the incidence of stent dysfunction rate. **Appendix file 7.** the forest map of the incidence of morbidity. **Appendix file 8.** the forest map of the incidence of Postoperative Pancreatic Fistula (POPF). **Appendix file 9.** Assessment of risk of bias. **Appendix file 10.** PRISMA 2009 flow diagram of literature screening. **Appendix file 11.** Basic characteristics and quality assessment of enrolled documents

## Data Availability

All the data comes from databases. The author has sorted out all the data and attached to the attachment at the end of the article.

## Abbreviations

PBD: Preoperative biliary drainage
MBO: Malignant biliary obstruction
ENBD: Endoscopic nasobiliary drainage
EBS: Endoscopic biliary stenting
PTBD: Percutaneous transhepatic biliary drainage
POPF: Postoperative pancreatic fistula
